# Investigating the Reciprocal Interrelationships among the Ruminal Microbiota, Metabolome, and Mastitis in Early Lactating Holstein Dairy Cows

**DOI:** 10.3390/ani11113108

**Published:** 2021-10-30

**Authors:** Shih-Te Chuang, Kuan-Yi Li, Po-Wen Tu, Shang-Tse Ho, Cheng-Chih Hsu, Jui-Chun Hsieh, Ming-Ju Chen

**Affiliations:** 1 Department of Veterinary Medicine, College of Veterinary Medicine, National Chung Hsing University, Taichung 402204, Taiwan; stchuang@dragon.nchu.edu.tw; 2 Department of Animal Science and Technology, National Taiwan University, Taipei 106037, Taiwan; doraemon102267@gmail.com (K.-Y.L.); ray0983631720@gmail.com (P.-W.T.); jchsieh@ntu.edu.tw (J.-C.H.); 3 Department of Wood Based Materials and Design, National Chiayi University, Chiayi 600355, Taiwan; stho@mail.ncyu.edu.tw; 4 Department of Chemistry, National Taiwan University, Taipei 106319, Taiwan; ccrhsu@ntu.edu.tw

**Keywords:** ruminal microbiota, metabolome, mastitis, Holstein dairy cows

## Abstract

**Simple Summary:**

Dairy cow mastitis is an inflammatory disease often caused by bacterial infections. In the present study, we identified the ruminal microbial biomarkers and metabolites of mastitis in dairy cows. The investigation of the reciprocal interrelationships among the ruminal microbiota, metabolome, and mastitis revealed that short-chain fatty acid (SCFA)-producing microflora and the metabolites related to anti-inflammation and antibacterial activity were significantly higher in healthy cows than in those with mastitis. The identified potential species and metabolites might provide a novel perspective to assist in targeting the ruminal microbiota with preventive/therapeutic strategies against mastitis in the future.

**Abstract:**

Mastitis in dairy cow significantly affects animal performance, ultimately reducing profitability. The reciprocal interrelationships among ruminal microbiota, metabolome, and mastitis combining early inflammatory factors (serum proinflammatory cytokines) in lactating dairy cows has not been explored, thus, this study evaluated these reciprocal interrelationships in early lactating Holstein dairy cows to identify potential microbial biomarkers and their relationship with ruminal metabolites. The ruminal fluid was sampled from 8 healthy and 8 mastitis cows for the microbiota and metabolite analyses. The critical ruminal microbial biomarkers and metabolites related to somatic cell counts (SCC) and serum proinflammatory cytokines were identified by the linear discriminant analysis effect size (LEfSe) algorithm and Spearman’s correlation analysis, respectively. The SCC level and proinflammatory cytokines positively correlated with *Sharpea* and negatively correlated with *Ruminococcaceae UCG-014*, *Ruminococcus flavefaciens*, and *Treponema saccharophilum*. Furthermore, the metabolites xanthurenic acid, and 1-(1H-benzo[d]imidazol-2-yl) ethan-1-ol positively correlated with microbial biomarkers of healthy cows, whereas, xanthine, pantothenic acid, and anacardic acid were negatively correlated with the microbial biomarkers of mastitis cows. In conclusion, *Ruminococcus flavefaciens* and *Treponema saccharophilum* are potential strains for improving the health of dairy cows. The current study provides a novel perspective to assist in targeting the ruminal microbiota with preventive/therapeutic strategies against inflammatory diseases in the future.

## 1. Introduction

Calving and lactation are common events that often result in inflammation in dairy cows [1], leading to local and systemic signs of illness, such as uterine diseases and mastitis [2]. Approximately 30 to 45% of postpartum cows develop some type of inflammation in the first 30 to 60 days of lactation [3,4], for example, dairy cow mastitis is caused by bacterial infection of the mammary gland [5]. Prevention and therapeutic strategies that mainly depend on antibiotics are not currently effective, often leading to chronic and recurrent infections [6].

Dysbiosis in gastrointestinal microbiota is characterized as the loss of commensals/keystone taxa or diversity [7,8] and may cause mastitis because the composition and activity of the indigenous microbiota in the gastrointestinal tract directly affect the health of dairy cows through the production of metabolites, competition for nutrients, and immune system regulation [9]. The species related to mastitis in the udder, including *Staphylococcus aureus*, *Streptococcus* spp., and *Escherichia coli*, have also been identified in the intestine of mastitic cows [10,11]. Moreover, fecal material transplanted from diseased but not healthy cows caused mastitis in germ-free mice [11], thus, the gastrointestinal microbiota may play a crucial role in inflammation outside the gastrointestinal tract.

The rumen in dairy cows favors microbial growth and is important for digestion in the gastrointestinal system. Studies of diet [12], fermentation efficiency [13], milk production, and feed efficiency [14] suggest that ruminal microbiota play a crucial role in growth and production performance. Additionally, the ruminal microbiota in dairy cows has different levels of somatic cell counts (SCC) [15] and the abundance of microflora and metabolites associated with inflammation were significantly changed in the rumen of mastitic cows [16], suggesting a link between mastitis and the ruminal microbiome in dairy cows.

The immune response is another potentially related factor of mastitis, which is also associated with the gastrointestinal microbiome [17]. Lactating cows with clinical mastitis and subclinical mastitis [18] showed a marked increase in interleukin (IL)-2, IL-6, IL-1β, and tumor necrosis factor (TNF)-α. Yuan et al. [19] reported that repeated subacute inflammatory challenges by TNF-α promoted metabolic disorders in lactating cows by disturbing homeostasis via alternation of microbiota. Hence, the proinflammatory cytokines, such as TNF-α and IL-6, as early inflammatory factors, could potentially affect the gastrointestinal microbiota of mastitic cows. However, there is a lack of information related to proinflammatory cytokines and ruminal microbiota of mastitic cows.

The comprehensive characterization of the ruminal microbiota and metabolome and their role in disease is important for the development of efficient diagnostic tools and therapeutic interventions [20,21]. Although a recent study has investigated the rumen microbiome structure and metabolites activity in dairy cows with mastitis, the reciprocal interrelationships between ruminal microbiota, metabolome, and early inflammatory factors in early lactating dairy cows with mastitis have not been explored. The bacterial biomarkers and co-occurrence patterns also remain unknown. Thus, this study analyzed the relationships between the ruminal microorganism profile, metabolites, and proinflammatory cytokines of early lactating dairy cows related to health and mastitis, identifying possible ruminal bacterial biomarkers and their relationship with ruminal metabolites.

## 2. Materials and Methods

### 2.1. Sample Collection

The present study involved 16 lactating Holstein dairy cows with an average age of 2.75 ± 0.71 years old in a similar milk production stage (the first 40–60 days of lactation) from the same commercial farm. All cows were fed with total mixed ration (TMR) and water ad libitum, milked twice per day, and under the same management. The cows were separated into the healthy group (HC) and mastitic group (MC) according to the previous studies [5,16,18], and further conducted microbiota and metabolite analyses. Ruminal fluid, blood, and milk samples were collected 2 h after morning feeding (4 h after morning milking) following previously described methods with slight modifications [22,23]. Briefly, ruminal fluid was sampled by the oral stomach tube technique conducted by a licensed veterinarian. The initial 100 mL of ruminal fluid was discarded to avoid saliva contamination. Subsequently, 100 mL of fluid was retained and filtered through four layers of cheesecloth, then, stored in a −80 °C freezer until analysis. Each sample was accurately weighed and freeze-dried. Blood samples were collected from the coccygeal vein using a vacutainer tube (BD Vacutainer^®^, Plymouth, Devon, England). Quarter milk samples were collected after discarding the initial 10 mL of milk to avoid contamination. All animal experimental protocols were reviewed and approved by the Institutional Animal Care and Use Committee of National Taiwan University (Approval No: NTU105-EL-00022).

### 2.2. Analyses of Somatic Cell Counts and Serum Cytokines

California mastitis test (CMT) was performed using a California mastitis test kit (ImmuCell Corp., Portland, ME, USA) on the farm for early detection of mastitis. The SCC of quarter milk samples was analyzed using a Fossomatic FC instrument (Foss Electric, Hillerød, Denmark) and 200,000 cells/mL was considered an optimal threshold level to distinguish milk secretion from mammary quarters with or without clinical mastitis [24]. TNF-α and IL-6 levels were measured using commercial enzyme-linked immunosorbent assay kits (Bovine TNF-alpha and IL-6 DuoSet ELISA, R&D system, Minneapolis, MN, USA) according to the manufacturer’s instructions.

### 2.3. Microbiota Analysis

Total genomic DNA was extracted from 20 mg of the freeze-dried ruminal fluid sample using phenol-chloroform with the bead-beating method [25]. Bacterial V3–V4 regions of 16S rRNA genes were amplified by primers 341F (5′-CCTAYGGGRBGCASCAG-3′) and 806R (5′-GGACTACNNGGGTATCTAAT-3′), and eukaryotic V9 regions of 18S rRNA genes were amplified by primers 1380F (5′-CCCTGCCHTTTGTACACAC-3′) and 1510R (5′-CCTTCYGCAGGTTCACCTAC-3′). Barcoded amplicons were analyzed and sequenced using the Illumina HiSeq 2500 PE250 platform. Paired-end reads were merged by the FLASH v1.2.7 software [26]. Quality filtering of the raw tags was performed under a specific quality-controlled process using QIIME v1.7.0 software (University of Colorado, Boulder, CO, USA) to obtain clean tags [27,28]. The tags were then compared with the reference Gold database using the UCHIME algorithm to detect and remove chimera sequences [29,30]. Sequences with ≥97% similarity were assigned to the same operational taxonomic units (OTUs) by the Uparse v7.0.1001 software [31]. Taxonomic annotation of the representative sequence for each OTU was performed using the Ribosomal Database Project classifier v2.2 against the Silva v128 database [32,33]. OTU abundance information was normalized by a standard sequence number, which corresponded to the sample with the least sequences. Subsequent analyses were performed using these normalized data. The alpha diversity (Chao1 richness estimator and Shannon’s diversity index) and partial least squares discriminant analysis (PLS-DA) were analyzed with QIIME v1.7.0 and R v3.6.1 software (Bell Laboratories, Murray Hill, NJ, USA). The false discovery rate (FDR) was applied to conduct multiple testing for the correction of the *p*-value by the Benjamini-Hochberg procedure. The linear discriminant analysis (LDA) effect size (LEfSe) algorithm was executed to identify differential enrichment of abundant taxa between groups (http://huttenhower.sph.harvard.edu/lefse/) (The Huttenhower Lab, Boston, MA, USA) [34]. The LEfSe algorithm first detects features with significant differential abundance in the class of interest using the non-parametric factorial Kruskal–Wallis sum-rank test. Subsequently, biological consistency was investigated by a set of pairwise tests among subclasses using the non-parametric (unpaired) Wilcoxon rank-sum test. Finally, LDA scores were used to estimate the effect size of each differentially abundant feature [34]. Microbial biomarkers with log-transformed LDA scores greater than 3 were retained for subsequent plotting.

### 2.4. Metabolite Analysis

Metabolite analysis was performed using 50 mg of freeze-dried powder of the ruminal fluids extracted in 500 μL of 70% methanol. The samples were vortexed and centrifuged at 3000× *g* for 5 min to remove insoluble particles. The methanolic extracts were filtered through a 0.22 μm filter and stored at −80 °C for subsequent analysis. HPLC-MS/MS analysis was performed using a Shimadzu LC-20A HPLC system (Shimadzu, Kyoto, Japan) coupled to a linear ion trap–Orbitrap mass spectrometer (LTQ Orbitrap Velos, Thermo Fisher Scientific, Waltham, MA, USA). First, 10 μL of the methanolic filtrate was analyzed with a Reprosil 100 C18 column (250 × 4.6 mm; 5 μm particle size; Dr. Maisch GmbH, Ammerbuch-Entringen, Germany) using a solvent gradient method with solvent A (0.1% formic acid in deionized water) and solvent B (0.1% formic acid in acetonitrile) and a flow rate of 1 mL/min. The split eluent was introduced into the mass detector at approximately 0.3 mL/min. The gradient program was as follows: 0–10 min, 0–10% B; 10–30 min, 10–60% B; 30–40 min, 60–100% B; 40–60 min, 100% B; 60–65 min, 100–0% B; 65–70 min, 0% B. Heated electrospray ionization was operated at 50 °C, with a capillary temperature of 275 °C, an ionization voltage of 3.5 kV and sheath gas flow rate of 35 L/min. The mass spectrometer was programmed in positive mode. The survey scans were acquired in the Orbitrap mass analyzer operating at 120,000 full width at half maximum. A mass range of 100 to 2000 *m*/*z* and collision energy of 25 V were used for the survey scans. The method was set to analyze the top 10 most intense ions from the survey scan, and dynamic exclusion was enabled for 15 s. Raw data were analyzed using Compound Discoverer v2.0 (Thermo Fisher Scientific). The false discovery rate (FDR) was applied to conduct multiple testing for the correction of the *p*-value by the Benjamini–Hochberg procedure. Orthogonal PLS-DA (oPLS-DA) were performed in MetaboAnalyst 5.0 (https://www.metaboanalyst.ca) (Xia Lab, McGill University, Montreal, QC, Canada) [35]. The metabolites have variable importance in projection (VIP) value > 1.0 and FDR-adjusted *p*-values < 0.05 represent a statistical significance compared to the healthy group. The ruminal compounds were annotated using the mzCloud database and defined as a mass at a specific retention time with the prior criteria of a fully matched state and distribution of all spectra. Metabolites involved in the Kyoto Encyclopedia of Genes and Genomes (KEGG) pathways were analyzed using the KEGG database.

### 2.5. Statistical Analysis

A nonparametric Mann–Whitney U test was executed for all phenotypic and next-generation sequencing (NGS) data to identify significant differences between groups. Bacterial networks were constructed by Spearman’s correlation analysis to clarify the correlation between biomarkers at the genus and species levels. Spearman’s correlation analysis was also applied to perform the correlation between the relative abundance of biomarkers and metabolites. All statistical analyses were performed by Statistical Analysis System v9.4 (SAS Institute Inc., Cary, NC, USA) and R software (Bell Laboratories).

## 3. Results

### 3.1. Healthy and Mastitic Cows Demonstrated Significant Differences in Proinflammatory Cytokines

**Figure 1:**
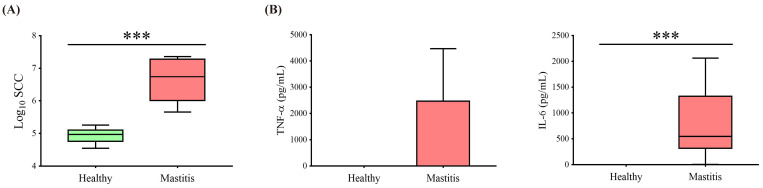
Somatic cell counts and serum cytokines of healthy and mastitic cows. (**A**) Log-transformed somatic cell counts and (**B**) serum cytokines (TNF-α and IL-6) were significantly higher in mastitic cows than in healthy cows (*** *p* < 0.001).

The mastitic cows were diagnosed by the combination of veterinary diagnosis, SCC (>200,000 cells/mL), and proinflammatory cytokines (IL-6 and TNF-α). Eight healthy cows were assigned to the HC group, and eight mastitis cows were assigned to the MC group. The SCC in the MC group was significantly higher than in the HC group (*p* < 0.05) (Figure 1A), with the MC group also having significantly higher IL-6 (*p* < 0.05) and showing a tendency for increased TNF-α (Figure 1B).

### 3.2. Healthy and Mastitis Cows Demonstrated Significant Differences in Ruminal Microbiota

**Figure 2:**
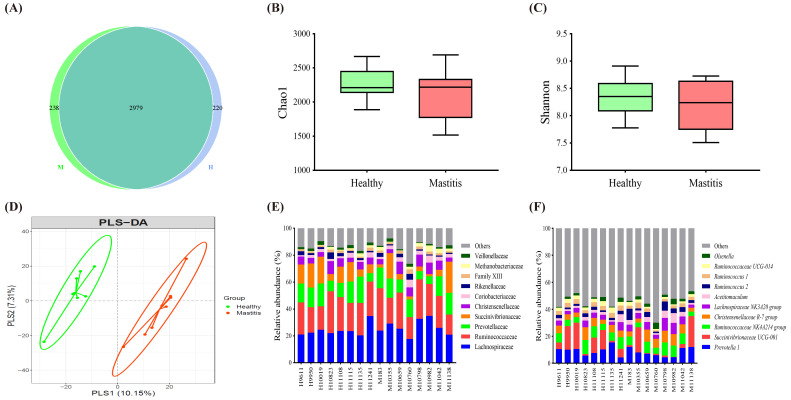
Ruminal bacteria and archaea composition identified by 16S rRNA sequencing of healthy and mastitic cows. (**A**) Venn diagram illustrating 2979 OTU of core microbiota identified by 16S rRNA sequencing of healthy (H) and mastitic (M) cows. (**B**) The Chao1 richness estimator and (**C**) Shannon’s diversity index. (**D**) Partial least squares discriminant analysis (PLS-DA) plot based on the relative abundance of OTUs indicates a significantly different composition of healthy versus mastitic cows. Ellipses represent 95% confidence intervals for each group. The top 10 (**E**) families and (**F**) genera identified in cow ruminal fluid, each bar refers to an individual cow.

After confirming the physiological condition of the cows, we further investigated whether mastitis could affect the ruminal microbiota, including bacteria, archaea, protozoa, and fungi, using NGS. For ruminal bacteria and archaea, 16S rRNA analysis revealed a total of 911,390 effective tags from 1,322,502 raw paired-end reads. The Venn diagram in Figure 2A illustrated that 2979 OTU in all samples were shared among the groups, with 238 and 220 unique OTUs for the MC and HC groups, respectively. The alpha diversity, Chao1 richness estimator, and Shannon’s diversity index, showed no significant difference (*p* > 0.05) between the HC and MC groups (Figure 2B). The top 10 dominant taxa at the family and genus level, which covered 86 and 46% of total family and genus, respectively, were the same between groups but with varying proportions (Figure 2C,D). Further beta diversity analysis discriminated the HC and MC groups using the partial least squares discriminant analysis (PLS-DA) plot (Figure 2E). The alpha and beta diversity results indicated that mastitis did not affect the richness and diversity of ruminal bacteria and archaea in dairy cows but might alter the composition of ruminal microbiota.

**Figure 3:**
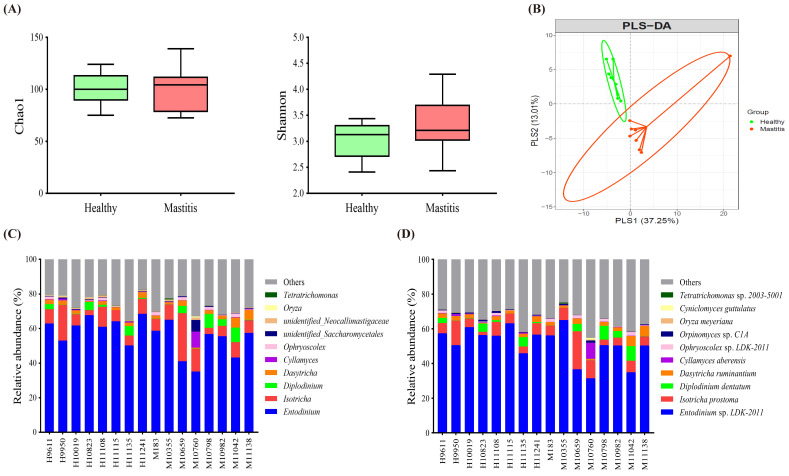
Ruminal protozoa and fungi composition were identified by 18S rRNA sequencing. (**A**) The Chao1 richness estimator and Shannon’s diversity index. (**B**) Partial least squares discriminant analysis (PLS-DA) plot based on the relative abundance of OTUs indicates a significantly different composition of healthy versus mastitis groups. Ellipses represent 95% confidence intervals for each group. The top 10 (**C**) genera and (**D**) species identified in cow ruminal fluid, each bar refers to an individual cow.

For ruminal protozoa and fungi, a total of 1,264,414 effective tags from 1,289,280 raw paired-end reads were obtained from 18S rRNA analysis. The Chao1 richness estimator and Shannon’s diversity index showed no significant difference (*p* > 0.05) between the HC and IC groups (Figure 3A). The PLS-DA plot illustrated a clear separation of the two groups (Figure 3B). Regarding the top 10 dominant taxa at the genus and species levels, the healthy cows had a significantly higher abundance of *Tetratrichomonas* sp. *2003–5001* (FDR adjusted *p* < 0.05) than the mastitic counterparts (Figure 3C,D).

### 3.3. Identification of the Critical Ruminal Bacterial Biomarkers Associated with Inflammation and Their Co-Occurring Patterns

**Figure 4:**
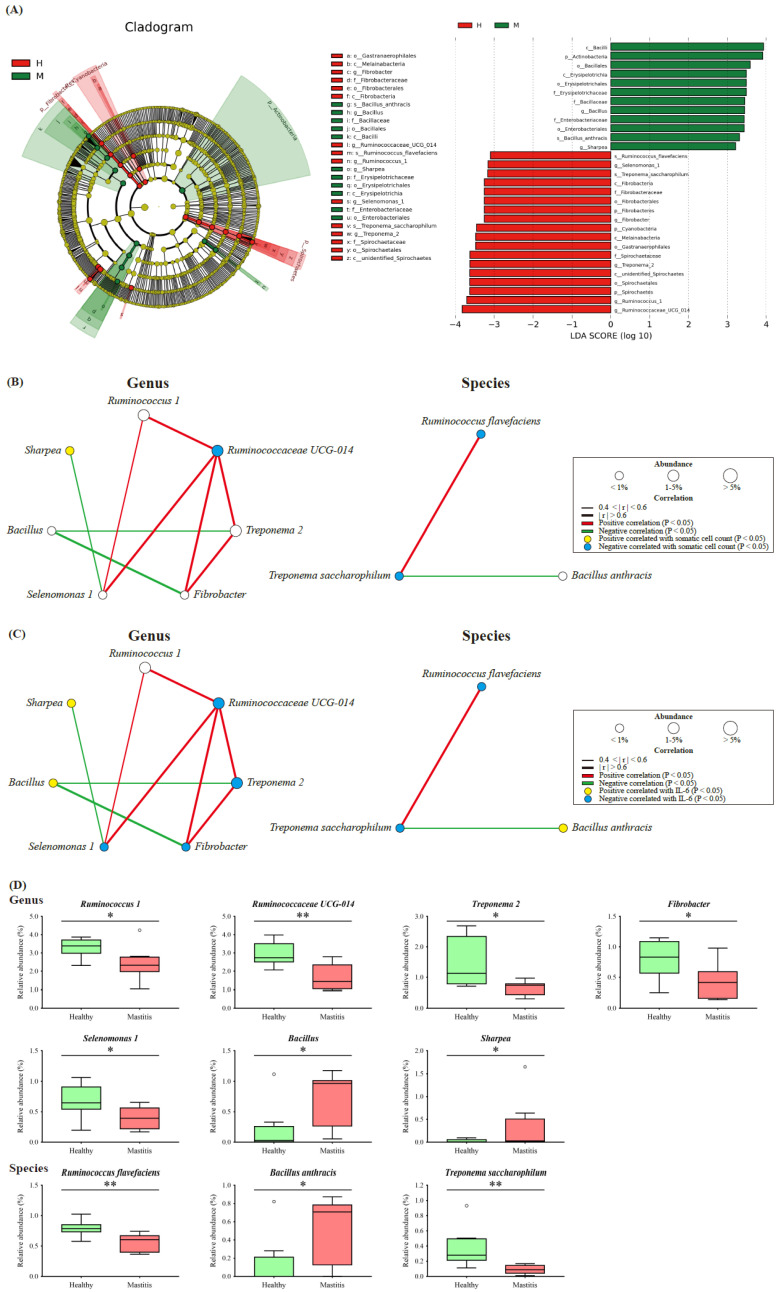
Significant differential biomarkers were identified using the linear discriminant analysis (LDA) effect size (LEfSe) algorithm. (**A**) LEfSe cladogram and log-transformed LDA scores illustrate differential enrichment of biomarkers between groups. Bacterial networks of ruminal microbiota show the correlations between the seven genus and three species with (**B**) SCC and (**C**) IL-6. Each node represents a biomarker and the size corresponds to its relative abundance. Yellow and blue nodes show significant positive and negative correlations with somatic cell count/IL-6 (*p* < 0.05), respectively. Red and green edges indicate significant positive and negative correlations between biomarkers (*p* < 0.05), respectively. (**D**) The significant relative abundance of differential biomarkers. Symbols indicate significantly different relative abundances between groups using the Mann–Whitney U test (* *p* < 0.05; ** *p* < 0.01).

Since the NGS results indicated that HC and MC could be distinguished by ruminal bacteria and archaea, in addition to dominant taxa, the less abundant but critical taxa associated with mastitis were identified as bacterial biomarkers by the LEfSe algorithm, revealing thirty influential taxonomic clades, including seven genera and three species (Figure 4A). In the HC group, the critical biomarkers were the genera *Ruminococcus 1*, *Ruminococcaceae UCG-014*, *Treponema 2*, *Fibrobacter*, and *Selenomonas 1*, as well as the species *Ruminococcus flavefaciens* and *Treponema saccharophilum*. The genera *Bacillus* and *Sharpea* and species *Bacillus anthracis* were the critical taxa in the MC group.

The co-occurrence patterns of the biomarkers were determined by constructing a bacterial network of the seven genera and three species, which was further correlated with the SCC and IL-6 levels. The SCC level positively correlated with *Sharpea* and negatively correlated with *Ruminococcaceae UCG-014*, *Ruminococcus flavefaciens*, and *Treponema saccharophilum* (*p* < 0.05) (Figure 4B). Among the genus bacterial network, *Sharpea* negatively correlated with *Selenomonas 1* (*p* < 0.05), whereas, *Ruminococcaceae UCG-014* positively correlated with *Ruminococcus 1*, *Treponema 2*, *Fibrobacter*, and *Selenomonas 1* (*p* < 0.05), indicating the critical role of *Ruminococcaceae UCG-014* in the network. In terms of the species bacterial network, *Ruminococcus flavefaciens* and *Treponema saccharophilum* were positively correlated (*p* < 0.05) (Figure 4B). IL-6 positively correlated with *Sharpea* and *Bacillus* and negatively correlated with *Ruminococcaceae UCG-014*, *Treponema 2*, *Fibrobacter*, *Selenomonas 1*, *Ruminococcus flavefaciens*, and *Treponema saccharophilum* (*p* < 0.05) (Figure 4C).

The relative abundance of the bacterial biomarkers associated with mastitis was consistent with the above findings (Figure 4D), with the mastitis group showing a significantly higher relative abundance of *Sharpea* and lower relative abundances of *Ruminococcaceae UCG-014*, *Ruminococcus flavefaciens*, and *Treponema saccharophilum* (*p* < 0.05) (Figure 4D).

### 3.4. Metabolomics Analysis Revealed Alterations in Ruminal Metabolites during Mastitis

**Figure 5:**
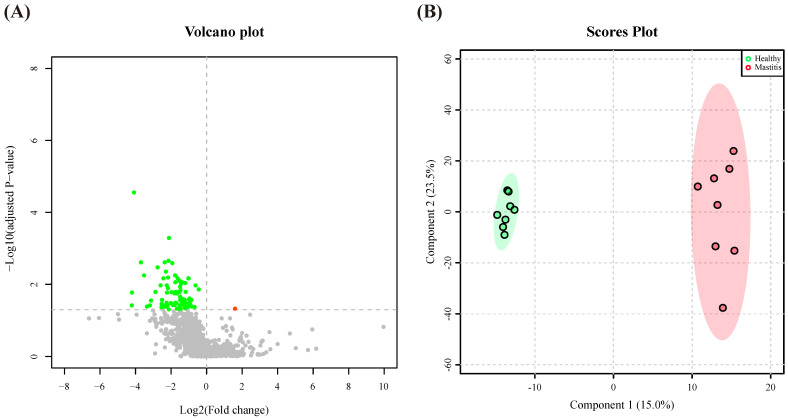
Different compositions of ruminal metabolites of the healthy and mastitic cows. (**A**) Volcano plot of 1336 compounds with log-transformed adjusted *p* values and fold change. The green and red dots indicate significantly higher metabolites in the healthy and mastitic groups, respectively. (**B**) Orthogonal partial least squares discriminant analysis (oPLS-DA) plot based on the 1336 compounds indicates significantly different metabolite compositions of the healthy and mastitic groups. Ellipses represent 95% confidence intervals for each group. Every dot represents a single individual cow.

Alterations in the microbiota could affect the metabolites in the rumen. Subsequently, the ruminal metabolites were investigated using a nontargeted metabolomics approach. In total, 3144 practicable peaks were obtained from the rumen fluid samples, and 1336 compounds were observed. A volcano plot representing the metabolite profile by plotting statistical significance (AIP > 1, FDR adjusted *p* < 0.05 threshold) against fold change indicated that the most ruminal metabolites with significant differences between the two groups were downregulated when the cows suffered from mastitis (Figure 5A). Moreover, the PLS-DA plot demonstrated a clear separation between the HC and MC groups, indicating a different ruminal metabolite composition (Figure 5B). After identifying the metabolites through blasting with the full match in the mzCloud online database, 84 metabolites were identified, of which 7 (AIP > 1, FDR adjusted *p* < 0.05) were significantly lower in the MC group than in the HC group (Table 1).

### 3.5. Reciprocal Interrelationships among the Ruminal Microbiota, Metabolome, and Inflammatory Cytokines

**Figure 6:**
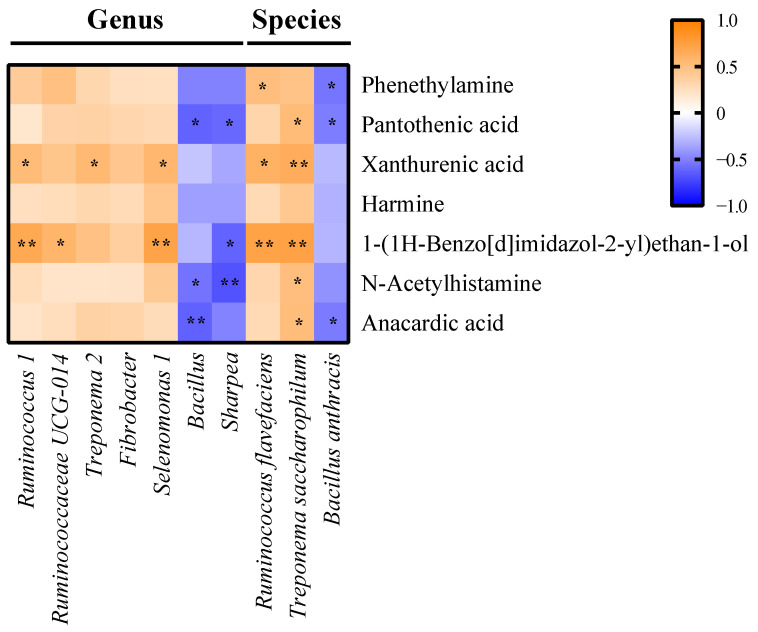
Reciprocal interrelationships between the ruminal microbiota and metabolome by Spearman’s correlation test shown at the genus and species levels. Orange and blue colors indicate positive and negative correlation coefficients, respectively. Symbols indicate the significant correlation between metabolites and biomarkers (* *p* < 0.05; ** *p* < 0.01).

The reciprocal interrelationships between ten ruminal biomarkers, seven differential metabolites, and inflammatory cytokines were analyzed by Spearman’s correlation test. Seven metabolites were significantly correlated with some of the critical bacterial biomarkers (*p* < 0.05) (Figure 6). The bacterial biomarkers in the HC group, including the genera *Ruminococcus 1*, *Ruminococcaceae UCG-014*, *Treponema 2*, and *Selenomonas 1*, as well as the species *Ruminococcus flavefaciens* and *Treponema saccharophilum*, positively correlated with xanthurenic acid, and 1-(1H-benzo[d]imidazol-2-yl) ethan-1-ol (*p* < 0.05) (Figure 6), which are related to tryptophan metabolism (Table 1). The genera *Bacillus* and *Sharpea* and species *Bacillus anthracis*, the bacterial biomarkers in the MC group, negatively correlated with xanthine, pantothenic acid, and anacardic acid (*p* < 0.05) (Figure 6), which are associated with purine metabolism and pantothenate and CoA biosynthesis (Table 1).

## 4. Discussion

In the present study, 16S rRNA and 18S rRNA analysis based on a single-molecule sequencing platform was employed to profile the taxonomic structure of bovine rumen microbiota and determine differences between healthy cows and inflammatory cows with clinical mastitis in the early lactating period (40–60 days). A mastitis-associated change in the microbiota in milk, characterized by a general increase in *Enterococcus*, *Streptococcus*, and *Staphylococcus* [36], was also observed in the rumen of the MC group, suggesting that alteration of the ruminal microbiota could be associated with bovine mastitis. Another study found that the abundance of microflora associated with inflammation was significantly different in the rumen of mastitis cows [16], providing additional support for our finding.

Dysbiosis of the ruminal microbiota might play an essential role in increasing susceptibility to mastitis, so targeting the ruminal microbiota is a potential therapy. The microbiota alpha diversity results, Venn diagram, and the relative abundance of the taxa at the different levels based on both 16S rRNA and 18S rRNA sequencing illustrated that the core microbiota remained stable and resilient regardless of the differences in mastitis. Some core bacterial taxa identified during early lactating cows with mastitis were consistent with those identified in dry period cows [37], lactating dairy cows [38,39], and beef cattle [40]. The genera *Prevotella*, *Ruminococcus*, and *Butyrivibrio*, which are the most abundant in the rumens of lactating cows and dry period cows [38], were the predominant genera in the MC and HC groups. These genera are involved in various ruminal functions, including the breakdown of fibrous plant material to generate SCFA [1,31], protein degradation, lipid biohydrogenation [32], and microbial inhibitor production [33,34]. Wang et al. [16] observed a higher abundance of *Prevotella_1* in ruminal microbiota in healthy cows compared to mastitic cows. These genera could be considered as part of health ruminal core microbiota and play important roles in maintaining cow health.

Although the ruminal microbiota in the MC and HC groups possessed similar core microorganisms, the variation in ruminal microflora composition could effectively separate the groups by PCA plot and unweighted UniFrac. Thus, key microorganisms associated with two groups were identified using LEfSe. Through additional Spearman correlations, five genera and two species were identified as the critical ruminal bacterial biomarkers associated with healthy cows, and these biomarkers negatively correlated with SCC and IL-6. The genus *Ruminococcus*, including *Ruminococcus* 1 and *Ruminococcaceae UCG-014*, and the species *Ruminococcus flavefaciens*, biomarkers in the HC group, were identified as the second most predominant core taxon in the present study. Studies have indicated that ruminal *Ruminococcus* could break down fibrous plant material to generate acetate, formate, succinate, and other SCFA [41,42,43]. *Ruminococcus 1* is also associated with thiamine synthesis [44]. The other three biomarkers in the HC group, including genera *Fibrobacter*, *Selenomonas,* and *Treponema*, are all SCFA producers. *Fibrobacter*, an important cellulolytic bacterium, digests fiber in the rumen to produce succinate, acetate, and formate [45]. *Selenomonas* generates propionate via decarboxylate succinate produced by *Fibrobacter*, indicating that both genera had interspecies interactions in the rumen ecosystem [46]. The genus *Treponema* and species *Treponema saccharophilum* ferment pectin to produce acetate as a major endproduct [47]. This genus has been reported to be negatively associated with IL-1β mRNA expression [48], revealing its potential anti-inflammatory effect, which is paralleled by our findings. Additionally, the protective effects of SCFAs against mastitis have been intensively studied, including decreasing rumen epithelium lipopolysaccharide (LPS) levels [49], inhibiting the production of proinflammatory cytokines and the activation of the NF-κB signaling pathway [49,50], and protecting against LPS-induced mastitis by inhibiting histone deacetylases [50,51]. Although bacterial enumeration from sequencing data is difficult to extrapolate the ruminal SCFA concentrates, the SCFA-producing microflora identified in the present study may be crucial for preventing mastitis and merit further investigation.

Conversely, two genera and one species were the critical biomarkers related to inflammatory cows and were positively correlated with SCC and IL-6. *Sharpea*, a biomarker in the MC group, has been reported to participate in lactate production and utilization, which was increased by a grain-based subacute ruminal acidosis (SARA) challenge [52,53]. *Bacillus* and *Bacillus anthracis* were other biomarkers identified in the MC groups. Although partial *Bacillus* in the gastrointestinal tract is harmless, *Bacillus anthracis* is an obligate pathogen, which could cause severe breast infection in lactating cows [54]. In general, these biomarkers significantly upregulated in the rumen of the MC group have been described with certain pathogenicity, but their pathogenicity in the rumen of cows requires further investigation. Moreover, this finding also suggests that specific taxa in the rumen, not necessarily the dominant microorganisms, significantly affect the inflammatory status of early lactating cows.

In addition to microbiota differences, the levels of seven rumen fluid metabolites also significantly changed between healthy and mastitic cows. These differential metabolites may be potential biomarkers for the diagnosis of mastitic cows. The analysis of the correlation between rumen microbial biomarkers and metabolites associated with SCC and inflammatory cytokines revealed that xanthurenic acid, and 1-(1H-benzo[d]imidazol-2-yl) ethan-1-ol positively correlated with microbial biomarkers of healthy cows. These metabolites, related to the tryptophan metabolic pathway, have been reported to induce anti-inflammatory responses via the reduction in IFN-γ to improve immunity in animals [55,56]. Conversely, xanthine, pantothenic acid, and anacardic acid negatively correlated with the microbial biomarkers of mastitis cows. Pantothenic acid is necessary for a variety of metabolic reactions because of its incorporation into coenzyme A and acyl-carrier-protein [57]. Anacardic acids have a high antioxidant capacity associated with the inhibition of superoxide generation and xanthine oxidase [58]. This compound also possessed antibacterial activity against methicillin-resistant *Staphylococcus aureus*, which mainly causes mastitis in dairy cows [59]. Several other differential metabolites that were previously unreported or with unknown function in the rumen related to inflammation remain to be clarified. The present results suggest that the metabolites generated by ruminal microbiota play a critical role in maintaining the health of dairy cows and developing mastitis. However, it is of note that none of the critical ruminal metabolites identified in the HC and MC groups were the same in a previous study of rumen metabolites in dairy cows with mastitis [16]. This could be explained by the differences in diet and growth environment, which could also affect the ruminal microbiota and metabolites. Additionally, the inflammation could be a factor influencing the feed intake, which might also affect the microbiota. The effect of diet, feed intake, and environment on the microbiota and metabolome in lactating cows with mastitis should be further investigated.

## 5. Conclusions

In conclusion, this study systematically identified the profile of the ruminal microbiota and metabolome and elucidated distinct differences between healthy and mastitic cows in the early lactating period. Healthy cows possessed biomarkers associated with SCFA-producing bacteria and generated metabolites associated with anti-inflammation, antioxidation, and antibacterial activity. The microbiota of mastitic cows was characterized by a decreased prevalence of SCFA-producing bacteria. The current study provides new insight into mastitis in cows by identifying the critical ruminal bacterial biomarkers and potential ruminal metabolites and by investigating the reciprocal interrelationships between the ruminal microbiota and metabolome. Two species, *Ruminococcus flavefaciens*, and *Treponema saccharophilum*, were identified as HC biomarkers, hence merit further study. These findings provide a novel perspective to assist in targeting the ruminal microbiota for preventive strategies against inflammatory diseases in the future.

## Figures and Tables

**Table 1:** Characterization of differential ruminal metabolites in healthy and mastitic cows by HPLC-MS/MS.

Metabolites	Mean Intensity		VIP ^1^	FC ^2^	*p*-Value	FDR ^3^	KEGG Pathway ^4^
	Healthy	Mastitic					
Phenethylamine	152891.87	100793.32	2.14	−0.60	0.0002	0.0107	Phenylalanine metabolism
Pantothenic acid	114575.33	33733.69	1.94	−1.76	0.0050	0.0671	Pantothenate and CoA biosynthesis
Xanthurenic acid	63550.91	30866.76	2.12	−1.04	0.0024	0.0423	Tryptophan metabolism
Harmine	55696.01	31729.60	1.59	−0.81	0.0107	0.0949	Biosynthesis of alkaloids derived from shikimate pathway
1-(1H-Benzo[d]imidazol-2-yl)ethan-1-ol	42332.18	20576.09	2.28	−1.04	0.0001	0.0068	NA
N-Acetylhistamine	25705.04	5040.48	1.79	−2.35	0.0056	0.0689	Histidine metabolism
Anacardic acid	9944.58	1930.33	1.66	−2.37	0.0032	0.0505	NA

^1^ VIP: Variable importance in projection. ^2^ FC: log2(fold change); Fold change (ratio in the log base 2 scale) between the mastiticand healthy groups. ^3^ FDR: False discovery rate. ^4^ NA indicates a metabolite not associated with the KEGG pathway.

## Data Availability

The datasets used and/or analyzed in the current study are available from the corresponding author on reasonable request.
